# Exposition to Biological Control Agent *Trichoderma stromaticum* Increases the Development of Cancer in Mice Injected With Murine Melanoma

**DOI:** 10.3389/fcimb.2020.00252

**Published:** 2020-05-29

**Authors:** Uener R. dos Santos, Marliete C. Costa, Gustavo J. C. de Freitas, Flávia S. de Oliveira, Bianca R. Santos, Juneo F. Silva, Daniel A. Santos, Adriana A. M. Dias, Luciana D. de Carvalho, Danillo G. Augusto, Jane L. dos Santos

**Affiliations:** ^1^Departamento de Ciências Biológicas, Universidade Estadual de Santa Cruz, Ilhéus, Brazil; ^2^Departamento de Microbiologia, ICB – Universidade Federal de Minas Gerais, Belo Horizonte, Brazil; ^3^Departamento de Genética, Ecologia e Evolução – ICB, Universidade Federal de Minas Gerais, Belo Horizonte, Brazil; ^4^Programa de Pós-Graduação em Genética, Universidade Federal Do Paraná, Curitiba, Brazil

**Keywords:** biological control, *Trichoderma*, cancer, metastasis, patter recognition receptor, Tricovab

## Abstract

Biological control agents (BCA) are an alternative to chemical pesticides and an emerging strategy to safely eliminate plant pathogens. *Trichoderma* spp. are the most common fungi used as BCAs. They produce spores that are released into the air and can potentially interact with immune system of mammals. We previously showed that *Trichoderma* affects expression of genes encoding pattern recognition receptors (PRRs) and cytokines in mice. PRRs are involved in the recognition of microorganisms and can lead to pro-tumoral signaling. Here, we evaluated if mice injected with low doses of murine melanoma exhibited increased development of lung tumor when treated with conidia of *T. stromaticum*. Mice treated with *T. stromaticum* and inoculated with B16-F10 melanoma cells exhibited significant increase in tumor uptake (*p* = 0.006) and increased number of visible nodules in the lungs (*p* = 0.015). We also analyzed mRNA expression levels of genes encoding PRRs in lung of mice exposed to *T. stromaticum* and demonstrated that mice treated with *T. stromaticum* conidia exhibited lower expression levels of *Clec7a* and increased expression of *Tlr4* (toll like receptor 4) compared to non-treated controls. The expression levels of *Clec7a* and *Tlr2* were increased in mice treated with *T. stromaticum* and inoculated with murine melanoma compared to controls only inoculated with melanoma. Our results demonstrate that intranasal exposition to *T. stromaticum* increases tumor in the B16-F10 model, which may raise concerns regarding the safety of its use in agriculture.

## Introduction

Biological control agents (BCAs) are an alternative to chemical pesticides for enhanced agricultural production (Verma et al., 2007). BCAs are composed of antagonistic microorganisms such as fungi or bacteria, which act against plant pathogens by lowering density of the phytopathogens in the field (Chet and Inbar, 1994; Syed Ab Rahman et al., 2018).

*Trichoderma* spp. are the most common antagonistic microorganism used as BCAs, comprising 60% of BCA used worldwide (Verma et al., 2007). The species *T. harzianum, T. koningii, T. longibrachiatum, T. asperelloides* and *T. stromaticum* are widely used worldwide. In Brazil, *T. stromaticum* is extensively applied to eliminate the witches' broom disease (*Moniliophthora perniciosa*) in Cacao (*Theobroma cacao*) plantations (Medeiros et al., 2010) under the comercial name Tricovab®. Although most *Trichoderma* fungi are non-pathogenic, they may represent potential risk to human health and cause opportunistic infections, particularly in immunocompromised and immunosuppressed individuals (Pomella et al., 2007; Lagrange-Xélota et al., 2008; Eduard, 2009; Sautour et al., 2018). *T. longibrachiatum* is the most commonly associated with opportunistic and invasive mycosis in humans, including peritonitis, lung and disseminated infections (Druzhinina et al., 2008).

Our research group has been focusing on the interaction between these fungi and the mammal immune system. We have previously demonstrated that exposition to conidia (asexually produced spores) of *T. asperelloides* and *T. stromaticum* by different routes can downregulate the immune system of mice. More specifically, we have shown their impact in peripheral blood, including inhibition of phagocytic ability in macrophages and changes in mRNA expression of genes encoding cytokines (Alves-Filho et al., 2011; dos Santos et al., 2017). In addition, we demonstrated that exposition to the conidia decreased the expression of genes encoding the pattern recognition receptors (PRRs) dectin-1, dectin-2, TLR (toll-like receptor) 2 and TLR4, which are critical for microbial recognition and pro-inflammatory response (Alves-Filho et al., 2011; dos Santos et al., 2017).

The effectiveness of the immune system is essential to control tumor growth, avoid the spread of cancer cells and the occurrence of metastasis (Erpenbeck et al., 2010; Eruslanov et al., 2014; Carpinteiro et al., 2015; Swierczak et al., 2015; Dasgupta et al., 2017). When tumor cells initially colonize the lung, a pro-inflammatory response induces differentiation of classically activated macrophages (M1) and type 1 T helper (Th1) cells, as well as activate T CD8+ response to control tumor growth (Altorki et al., 2019). However, immunosuppressed individuals are uncapable of building a robust immune response against tumor and other diseases, and exhibit higher risks of developing opportunistic infections and tumor progression (Carbone et al., 2014; Dropulic and Lederman, 2016; Manyam et al., 2017).

Several studies have demonstrated that toll-like receptors and others PRRs can affect tumor development and progression (Lowe et al., 2010; Zhang et al., 2011; Chiba et al., 2014; Dajon et al., 2019). Moreover, there is poor understanding of the real impact of exposition to BCAs for human health and their impact on the expression of genes encoding PRRs. We hypothesized that exposition to *T. stromaticum* can increase tumor growth and metastases and this could be partially explained by its impact on PPRs. We investigated the effect of intranasal exposition to *T. stromaticum* conidia in tumor development using a mouse model of pulmonary metastasis. Our results impressively show that *T. stromaticum* conidia significantly increase the risk of lung cancer in mice injected with metastatic melanoma cells.

## Materials and Methods

### Ethics Statement

C57BL/6 female mice (9–12 weeks) were acquired from Universidade Federal de Minas Gerais Animal Research Facility, maintained in specific pathogen-free conditions, with 12 h light/dark cycles receiving water and food *ad libitum*. The experiments with animals were conducted according to institutional guidelines for animal ethics and were approved by institutional animal care and use committees of both Universidade Estadual de Santa Cruz and Universidade Federal de Minas Gerais, under approval numbers 020/18 and 285/2018, respectively.

### Culture of *Trichoderma stromaticum* Conidia and Inoculation

*Trichoderma stromaticum* was obtained from Tricovab® (Ceplac, Brazil accession #Ts3550), a biofungicide developed by the Brazilian government and commercially available for the control of *Moniliophthora pernicious* in cocoa plantations (Pomella et al., 2007). *Trichoderma stromaticum* was cultivated on potato dextrose agar (PDA) in Petri dishes at 28°C in the dark until observation of conidia (7–15 days). After sporulation, conidia were collected using 3 mL of sterile phosphate buffered saline 1 × (PBS) and the suspension of conidia was washed three times with PBS at 2200 × *g* for 10 min at 12°C. The spore concentration was calculated by using Neubauer chamber. For animal inoculation 1 × 10^5^ conidia were suspended in 20 μL of PBS and inoculated by intranasal route (i.n.) once per week.

### Tumor Cell Culture and Inoculation

B16-F10 cells (murine melanoma, ATCC CRL-6475) were cultured in Dulbecco's modified eagle's medium—DMEM (Gibco) supplemented with 10% of fetal bovine serum (Gibco), penicillin (100 U/mL), streptomycin (10 mg/mL), and L-glutamine (2 mM/mL) and cultured in a humidified incubator at 37°C with 5% of CO_2_ saturation. For the experimental metastasis model, B16-F10 cells (5 × 10^4^ cells) were suspended in 100 μL of PBS and injected intravenously (i.v.) into the lateral tail vein. The same cell preparation was used in all experiments.

### Experimental Design

The animals were divided into experimental groups with 5 to 6 mice per group. A first group of mice were intranasally treated only with *T. stromaticum* conidia (1 × 10^5^ conidia) once per week starting at day 0 for 4 weeks. A second group was injected only with a low dose of B16-F10 tumor cells (5 × 10^4^ cells) intravenously at day 9. For comparison, the control group did not receive any treatment.

To evaluate if a previous or posterior exposition to *T. stromaticum* conidia can be a risk factor and impact tumor development, two different protocols were designed. In protocol 1, a group of mice were treated with conidia (1 × 10^5^ conidia) once per week starting at day 0 during 4 weeks, and injected with B16-F10 cells (5 × 10^4^ cells) at day 9; for comparison a control group was treated only with PBS once per week (instead of conidia) and injected with B16-F10 cells at day 9. In protocol 2, a group of mice were injected with B16-F10 cells (5 × 10^4^ cells) at day 9 and treated with conidia (1 x 10^5^ conidia) once per week starting at day 10 during 3 weeks; for comparison a control group was injected with B16-F10 cells at day 9 and treated only with PBS one per week (instead of conidia).

The body weight was assessed every 2 days, from day 0 to day 24. At day 24 (15 days post tumor cell inoculation), all mice were euthanized with ketamine and xylazine (100 and 10 mg/kg) by intraperitoneal route (i.p.). The lungs were harvested and data from lung weight, lung-to-body weight ratio, tumor uptake were collected, and the number of visible metastatic nodules were counted. The right lung was used for histological analysis and the left lung was used for RNA extraction.

### Lung Histopathology

After euthanasia, the right lungs were collected, fixed in 10% buffered formalin, paraffin-embedded, then sectioned at 4 μm slices and stained with hematoxylin and eosin (H&E) for histopathological assessment. For tumor analyses we established a micrometastatic score as described below. The total micrometastatic score is the sum of the number of micrometastasis range from 0 to 3. The severity score is: none = 0; one to four = 1; five to eight = 2; more than eight = 3. The analysis was performed on three random fragments of each collected right lung.

### qRT-PCR

Total RNA from tissue was extracted using TRIzol® reagent method (Life technologies, USA Molecular Research Center, Inc). Subsequently, RNA quantification was performed using Nanodrop® ND-2000 (Thermo Scientific-USA) and two micrograms of total RNA were retro-transcribed with ImProm-II™ Reverse Transcriptase kit (Promega) and Oligo(dT)20 primers (IDT). qPCR was performed on QuantStudio3 Real-Time PCR System and *QuantStudio*™ *Design & Analysis* Software (*Applied Biosystems*, Life Technologies, EUA) using GoTaq® qPCR Master Mix System (Promega). Each cDNA sample was analyzed in duplicates with a total reaction volume of 10 μL, with 2 μL of cDNA samples as template (20 ng), 5 μL de Master Mix (2X) and 0.4 μL of each primer (200 ηM). The reactions were under the following conditions: 95°C for 10 min, 40 cycles of 95°C for 15 s, and 60°C for 60 s.

Primers sequences for genes *Rn18s, Clec7a, Tlr2* and *Tlr4* used in this study are given in Supplementary Table 1. All primers were validated previously (dos Santos et al., 2017). Expression values were normalized to the expression of *Rn18s*, encoding 18S ribosomal RNA, by the comparative method according to formula 2^−ΔΔCt^; ΔCt corresponds to the Ct value of the target gene subtracted from the Ct of the endogenous gene, and ΔΔCt corresponds to the ΔCt value of each condition subtracted from the control group ΔCt median (Livak and Schmittgen, 2001).

### Data Analysis

Statistical analysis was performed using GraphPad Prism Software version 7.0. The Kolmogorov–Smirnov test was used to test the normal distribution of the variables in all experiments before statistical analysis. The body weight change was analyzed by two-way ANOVA, followed by Bonferroni's post-test. The analysis between two groups was assessed using Mann-Whitney test for visible nodules and micrometastatic score, chi-square test for tumor uptake and Student's *t-*test for other analysis. Data are presented as mean ± standard error of mean (SEM) and values of *p* < 0.05 were considered for statistical significance.

## Results

### Conidia of *T. stromaticum* Significantly Increase Lung Tumor Uptake and Development

**Figure 1:**
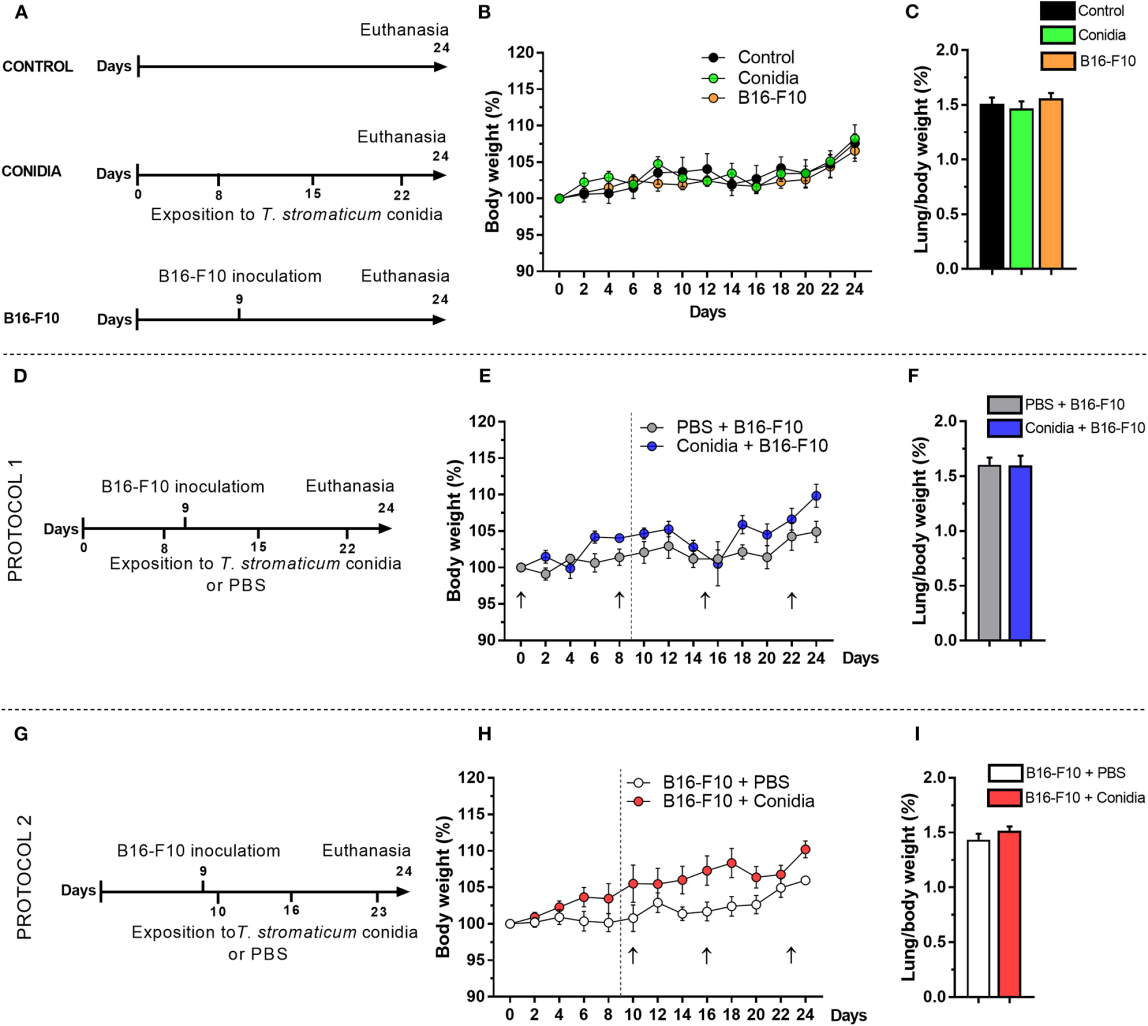
Effect of *T. stromaticum* conidia in mice from B16-F10 melanoma model. **(A)** Experimental design from groups conidia, B16-F10 and a control group (*n* = 6 mice per group); numbers in the horizontal bar indicate the days of inoculation of conidia (below) and days of tumor cell inoculation (above); **(B)** body weight kinetics; and **(C)** lung-to-body weight ratio. **(D)** Experimental design from Protocol 1 (*n* = 5–6 mice per group); **(E)** body weight kinetics; black arrow indicates the days of exposition to *T. stromaticum* conidia and dashed line the day of tumor cells inoculation; **(F)** lung-to-body weight ratio. **(G)** Experimental design from Protocol 2 (*n* = 5–6 mice per group); **(H)** body weight kinetics; black arrow indicates the days of exposition to *T. stromaticum* conidia and dashed line the day of tumor cells inoculation; **(I)** lung-to-body weight ratio. The body weight was compared between groups using Two-way ANOVA, followed by Bonferroni's post-test and Student's *t*-test for lung weight and lung-to-body weight ratio analysis. Data are presented as mean ± SEM. Value of *p* < 0.05 was considered for statistical significance.

A group of mice was initially exposed intranasally to inoculum of *T. stromaticum* and a second group was injected with a low dose of B16-F10 cells (Figure 1A, experimental design). No difference was observed in body weight kinetics and lung-to-body weight ratio compared control group (Figures 1B,C).

We used the melanoma metastatic model B16-F10 to evaluate the impact of the intranasal exposition to *T. stromaticum* conidia on the development of murine lung cancer. Considering that humans may be occupationally exposed to conidia of BCAs before or after tumor development, we tested two different protocols in mice, being the exposition to conidia before tumor cells inoculation in protocol 1 (Figure 1D, experimental design) and after tumor cells inoculation in protocol 2 (Figure 1G, experimental design). Significant difference was not observed in body weight kinetics (Figures 1E,H) and lung-to-body weight ratio in both protocols (Figures 1F,I).

**Figure 2:**
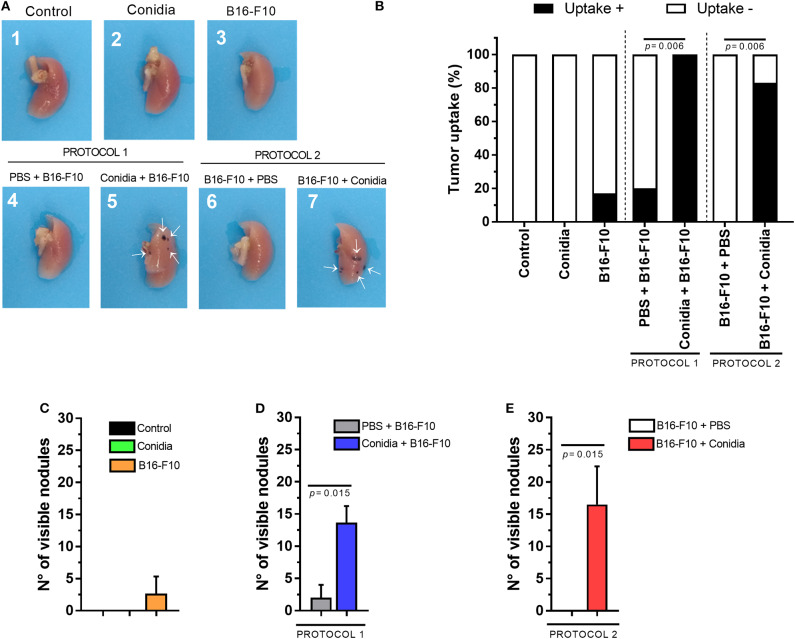
Conidia of *T. stromaticum* significantly increase lung tumor uptake and development. **(A)** Illustrative macroscopic image of the right lungs from mice in control group (1); inoculated only with conidia (2); only with B16-F10 (3); with PBS and B16-F10 (4); inoculated with conidia and B16-F10 cells (5); inoculated with B16-F10 cells and PBS (6); or inoculated with B16-F10 cells and conidia (7). White arrows (→) indicate the presence of tumor nodules. **(B)** Tumor uptake determined by the presence of tumor in the lung. Number of visible nodules count on lung surface from **(C)** mice treated only with conidia, only with B16-F10 cells and in control groups; in **(D)** Protocol 1 and **(E)** Protocol 2. The tumor uptake was assessed using Chi-square test and Mann-Whitney test for visible nodules. Data are presented as mean ± SEM (*n* = 5–6 mice per group). Value of *p* < 0.05 were considered for statistical significance.

The data show that mice treated only with conidia did not exhibit macroscopic alterations in the lung compared to control. In contrast, macroscopically tumor uptake was observed by the presence of multifocal dark pigmented small areas in pleural surface of the lung of mice treated with conidia before or after B16-F10 cells injection (Figure 2A). In group B16-F10, only one animal developed lung tumor at day 15 after inoculation of melanoma cells (1/6, 16.6%; Figure 2B) with reduced visible nodules in pleural lung surface (Figure 2C). In contrast, lung tumor uptake was observed in 6/6 (100%) and 5/6 (83%) of the mice in groups treated with conidia before or after injection of B16-F10 cells, respectively (protocol 1, *p* = 0.006; protocol 2, *p* = 0.006; Figure 2B). For comparison, in mice treated with PBS, only 1/5 (20%) and 0/5 (0%) of the implanted tumors metastasized and colonized the lungs to form visible tumor until 15 days post inoculation of B16-F10 cells in protocol 1 and 2, respectively (Figure 2B). No differences were observed between the groups injected with tumor cells (B16-F10, PBS+B16-F10 from protocol 1 and B16-F10+PBS from protocol 2; Supplementary Figure S1).

We observed increased number of visible nodules in the lungs of mice from group treated with conidia before injection of B16-F10 cells (13.67 ± 2.56) compared to control (2 ± 2) in protocol 1 *(p* = 0.015, Figure 2D). In protocol 2, the increased count in visible nodules was also observed from 0 ± 0 to 16.5 ± 9.95 nodules in the lung of mice treated with conidia compared to control (*p* = 0.015; Figure 2E). We also compared tumor uptake and visible nodules count from mice in both protocols. This analysis revealed no difference between protocols, indicating that prior or posterior exposure to *T. stromaticum* conidia impact tumor development equally (Supplementary Figure S2). Data from tumor in experimental mouse model are presented in Supplementary Table 2.

### Histopathological Characterization of Lung Tissue in B16-F10 Melanoma Model

**Figure 3:**
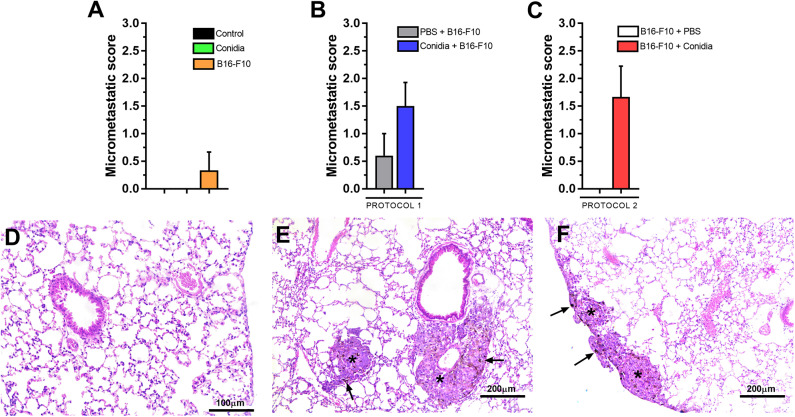
Histopathological analysis of mice in experimental model. **(A)** Micrometastatic score from mice treated with conidia, B16-F10 and control; **(B)** in protocol 1 and **(C)** protocol 2. **(D)** Photomicrograph of lung from mice challenged only with conidia. **(E)** Representative photomicrograph of early pulmonary micrometastasis in the subpleural and **(F)** periarteriolar area of the lung parenchyma from mice challenged with conidia and B16-F10. Asterisk (*) indicate micrometastatic foci and black arrow (→) indicate the presence of melanin in micrometastatic foci. Paraformaldehyde-fixed, paraffin-embedded sections were stained with hematoxylin & eosin (H&E). The micrometastatic score was assessed by Mann-Whitney test. Data are presented as mean ± SEM (*n* = 5–6 mice per group). Value of *p* < 0.05 were considered for statistical significance.

Mice treated only with melanoma cells exhibited low micrometastatic score (Figure 3A). We observed no difference in the micrometastatic score between lung of mice treated with conidia in protocol 1 (*p* = 0.245, Figure 3B) and in protocol 2 (*p* = 0.061, Figure 3C) compared to control. No differences between the controls or between protocols 1 and 2 for micrometastatic score were observed (Supplementary Figure S3).

We observed presence of tumor cell embolism in small vessels in mice with micrometastasis. Although no inflammatory infiltrate or endothelial reactivity was present, we observed presence of rare macrophages and neutrophils around micrometastatic foci. Representative photomicrographs of lung tissue from mice treated only with conidia, with absence of inflammatory infiltrate or histological alteration in lung parenchyma are shown in Figure 3D. Early pulmonary micrometastasis was found in the subpleural (Figure 3E) and periarteriolar (Figure 3F) areas of the lung parenchyma in mice treated with conidia and B16-F10 cells.

### Expression Levels of Pattern Recognition Receptors in Lung Are Affected by *T. stromaticum* Conidia

**Figure 4:**
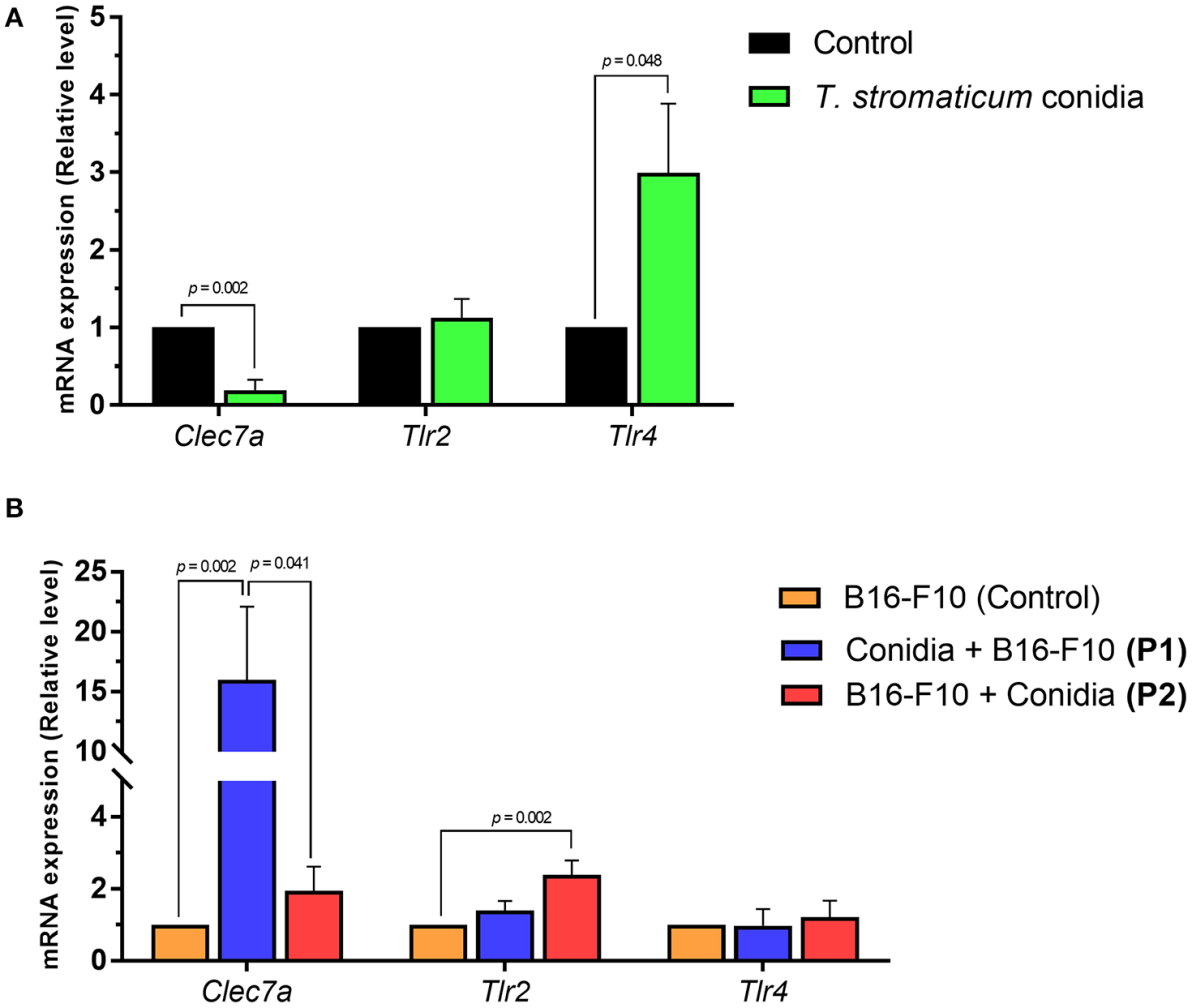
Expression levels of pattern recognition receptors. Abundance of mRNA transcripts of genes *Clec7a, Tlr2* and *Tlr4* from lungs tissue of mice. **(A)** Control and treated with *T. stromaticum* conidia. **(B)** Mice from protocols 1 (conidia + B16-F10) and protocol 2 (B16-F10 + Conidia) compared to control group only treated with B16-F10. Analyzed with Mann–Whitney test. Data are presented as mean ± SEM (*n* = 5–6 mice per group). Value of *p* < 0.05 were considered for statistical significance.

To test the expression of genes for PRRs in our model, we analyzed expression of genes encoding dectin-1 (*Clec7a*) and toll-like receptors (*Tlr2, Tlr4*) in lung tissue of mice exposed to *T. stromaticum* conidia in comparison to those not exposed. Mice treated only with *T. stromaticum* conidia presented lower mRNA expression levels of *Clec7a* (5.1-fold, *p* = 0.002) and increased expression of *Tlr4* (3-fold, *p* = 0.048; Figure 4A). In contrast, we observed no differences for *Tlr2* (Figure 4A).

We observed higher *Clec7a* mRNA expression levels in mice from protocol 1 compared B16-F10 (control) group (16-fold, *p* = 0.002), but no significant difference was observed for mice treated in protocol 2 compared to control (Figure 4B). Mice from protocol 2 exhibted increased mRNA levels of *Tlr2* compared to control group (2.4-fold, *p* = 0.002; Figure 4B). No differences were observed for *Tlr4* between the compared groups.

## Discussion

Previous studies have shown that chemical pesticides represent environmental risk and cause deleterious consequences for neurodevelopmental toxicity, immunotoxicity, respiratory diseases, and different types of cancer (Sanborn et al., 2002; Bonner et al., 2016; Mostafalou and Abdollahi, 2017; Daam et al., 2019). In contrast, BCAs are regarded as a safer alternative (Wang et al., 2004; Syed Ab Rahman et al., 2018) and have been successfully used worldwide (Pomella et al., 2007; Verma et al., 2007). However, the fact that the microorganisms used as BCAs may also interact with organisms other than those targeted for the biocontrol points to a potential risk, especially for those individuals who may be chronically exposed. So far, studies to understand the consequences of using BCAs to human health are scarce.

Here, we used the wild type mouse strain C57BL/6 to provide better understanding of the impact of exposition to *T. stromaticum* conidia in tumor development in the well-known murine B16 melanoma model (Overwijk and Restifo, 2000; Hwang et al., 2013; Foerster et al., 2018). Our results show that intranasal exposition to conidia of *T. stromaticum* increases tumor in our animal model. Disturbance of the mammalian respiratory system as well as occurrence of opportunistic infections caused by BCAs have been previously described, including for *Trichoderma* (Loeppky et al., 1983; Halpin et al., 1994; Madsen et al., 2007; Sautour et al., 2018). Human infections by *Trichoderma* were described in patients with pulmonary cancer, pericarditis, peritonitis, keratitis, leukemia, endocarditis, and others (Jaakkola et al., 2002; Kredics et al., 2003; Lagrange-Xélota et al., 2008; Tascini et al., 2016; Recio et al., 2019). In many cases, complications of these infections caused death (Jacobs et al., 1992; Kredics et al., 2003; Sandoval-Denis et al., 2014). Because many *Trichoderma* species are cosmopolitan soil-borne, agriculture is considered a potential source of emerging human infection by *Trichoderma* in immunocompromised and immunosuppressed individuals (Sandoval-Denis et al., 2014; Hatvani et al., 2019). However, our study is the first to demonstrate the implications of *Trichoderma* in cancer.

We observed that mice inoculated with a low dose of metastatic cells and treated intranasally with *T. stromaticum* conidia exhibited increased tumor uptake and increased number of visible lung tumors compared to controls only treated with metastatic cells, with presence of micrometastatic foci in lung tissue. In both protocols, mice exhibited increased risk of developing lung cancer when exposed to *T. stromaticum* conidia. Although there are reports of *Trichoderma* infections in cancer patients (Lagrange-Xélota et al., 2008; Sautour et al., 2018), there are no studies associating these infections with prognosis and/or course of disease. Different from chemical pesticides, for which type and duration of exposition are directly related to the risk for human health and the risk is lower for non-occupational exposure (Sabarwal et al., 2018), these assumptions cannot be extended to BCA due to the scarcity of studies and lack of direct demonstration.

In our study, we applied intranasally 10^5^ conidia in mice to evaluate their effect on lung tumor, however, the normal exposure levels to fungal conidia depends on the environment. Indoors, the levels rarely exceed 10^4^ conidia/m^3^, while outdoors, the levels are normally below to 10^5^ conidia/m^3^. In contrast, the levels in workplaces are normally higher and easily exceed 10^6^ conidia/m^3^ (Eduard, 2009). A previous study with immunosuppressed mice showed that exposition to higher amounts of *T. longibrachiatum* conidia (10^7^ CFU/animal) caused death in all treated animals, in contrast to lower exposition levels (10^4^ or 10^5^ CFU/animal) (Paredes et al., 2016). In contrast, we only studied immunocompetent mice and applied a low dose of *T. stromaticum* conidia (10^5^ conidia/animal). When treated only with *T. stromaticum*, all animals in our study did not show any alterations in body weight or lung-to-body weight ratio after 24 days, and also did not exhibit any histopathological alteration in the lungs. Similarly, in mice that were only treated with the low dose of melanoma, only 1 out 6 developed lung cancer. Interestingly, the concomitant presence of *T. stromaticum* conidia and B16-F10 cells significantly increased the development of lung cancer (6 out 6). Our results cannot reveal the mechanisms that explain why *T. stromaticum* increases tumor in individuals injected with a low dose of murine melanoma. It is also not clear if inhaled conidia will impact tumor as strongly as intranasal injection. However, our data suggest that presence of *T. stromaticum* results in a poorer immune response against cancer cells in mice challenged with the melanoma injection.

To date, only few studies analyzed the interaction between these fungi and mammalian immune system cells (Alves-Filho et al., 2011; Dos Santos et al., 201; Konstantinovas et al., 2017). Results from our group demonstrated that *Trichoderma* spp. reduces the mRNA expression of genes encoding toll-like receptor and c-type lectin receptor (CLR) in mice (Alves-Filho et al., 2011; dos Santos et al., 2017). TLR and CLR recognize microorganisms and tumor cells; they are also expressed on malignant cells, and their stimulation and/or inhibition are implicated in tumor development and metastasis (Chiba et al., 2014; Dajon et al., 2017, 2019). To provide insights of how *Trichoderma* may affect cancer, we investigated the gene expression levels of pattern recognition receptors involved in anti-fungal and anti-tumor immune responses in lung of the mice used in our experiments.

Analyzing RNA isolated from lung, we show that mRNA levels of *Clec7a* was 5.1 times lower in mice exposed to *T. stromaticum* conidia. *Clec7a* encodes the receptor dectin-1, which is expressed on the surface of dendritic cells and macrophages. Dectin-1 recognizes N-glycan structures from tumor cells, such as in B16 lineage, and activates IRF5 (interferon regulatory factor 5) pathway, among others, to activate natural killer cells to effectively eliminate neoplastic cells (Chiba et al., 2014). Our results also show that *Clec7a* levels were 16 times higher in mice treated with both melanoma and *T. stromaticum* in comparison to treated with only melanoma. It has been demonstrated that dectin-1 signaling in macrophages was associated with pancreatic carcinoma and peritumoral immune-tolerance by suppression of T cell immunogenicity (Daley et al., 2017). The altered expression of *Clec7a* in lung tissue of mice treated with *T. stromaticum* conidia allows us to speculate that dectin-1 may be involved in the increased tumor uptake and increased number of visible nodules in our model through mechanisms that are still not clear. A more modest increase (2.4-fold) was observed for *Tlr2* in protocol 2. It was reported that lung cancer patients exhibit higher serum levels of TLR (Zhang et al., 2020). In mice, carcinoma induces activation of myeloid cells via TLR2 to stimulate lung metastasis with expression of TNF-α (Kim et al., 2009). TLR2 can also activate the transcription factor Stat3, and trigger immunosuppressive response in B16-bearing mice (Yang et al., 2009).

In conclusion, our results demonstrate that intranasal exposition to *T. stromaticum* conidia increases the development of lung cancer in mice injected with murine melanoma, which could be related to altered expression of genes encoding PRRs. It is important to notice that intranasal injection of conidia is not the way that humans and other mammals are naturally exposed to BCAs, and our results do not allow the conclusion that inhaling *T. stromaticum* conidia from the air will cause cancer. However, despite the fact that our observations do not imply that the use of this BCA is not safe, its association with increased tumor in our animal model cannot be ignored. It is not clear whether or not *T. stromaticum* could disturb immune responses in high doses and/or increase the incidence of tumor in immunocompetent or those chronically exposed individuals. Therefore, further studies are necessary in order to decipher the mechanisms of interaction between these BCA and immune cells, as well to evaluate their possible implications in human health.

## Data Availability Statement

All datasets generated for this study are included in the article/Supplementary Material.

## Ethics Statement

The animal study was reviewed and approved by both the Universidade Estadual de Santa Cruz and Universidade Federal de Minas Gerais Institutional Animal Care and Use Committee, under protocol numbers 020/18 and 285/2018, respectively.

## Author Contributions

US and JSa conceived and designed the study. US, MC, GF, and AD conducted *in vivo* experiments. US and FO performed cell culture. US, BS, and JSi conducted histopathological preparation and analysis. US, FO, AD, and LC performed qRT-PCR. US, MC, AD, JSi, DA, JSa, and LC analyzed data. JSa, DS, and AD provided reagents. US, DA, MC, JSa, AD, and JSi drafted the manuscript. All authors contributed to manuscript revision, read and approval the submitted version.

## Conflict of Interest

The authors declare that the research was conducted in the absence of any commercial or financial relationships that could be construed as a potential conflict of interest.
