# Supplementary material for: Exposition to Biological Control Agent Trichoderma stromaticum Increases the Development of Cancer in Mice Injected With Murine Melanoma
Source: Front Cell Infect Microbiol. 2020 May 29;10:252. doi: 10.3389/fcimb.2020.00252 (PMC7272596; doi:10.3389/fcimb.2020.00252)
Supplement: Supplementary file 1 [file Table_1.pdf]

Supplementary Table 1. Primers sequences for the specific genes used in this study.

Genes	Primer Sequence		Reference
Rn18s	Forward:	5'-TTCGTATTGCGCCGCTAGA-3'	dos Santos, A. G., Mendes, É. A., de Oliveira, R. P., Faria, A. M. C., de Sousa, A. O., Pirovani, C. P., et al. (2017). Trichoderma asperelloides Spores Downregulate dectin1/2 and TLR2 Receptors of Mice Macrophages and Decrease Candida parapsilosis Phagocytosis Independent of the M1/M2 Polarization. *Front. Microbiol.* 8, 1–13. doi:10.3389/fmicb.2017.01681.
	Reverse:	5'-CTTTCGCTCTGGTCCGTCTT-3'	
Clec7a	Forward:	5'-GAACCACAAGCCCACAGAAT-3'	
	Reverse:	5'-CATGGCCCTTCACTCTGATT-3'	
Tlr2	Forward:	5'- GTGGTACCTGAGAATGATGTGGG-3'	
	Reverse:	5'- GTTAATTAAGTCAGGAACTGGGTG-3'	
Tlr4	Forward:	5'-CTGGGTGAGAAATGAGCTGG-3'	
	Reverse:	5'-GATACAATTCCACCTGCTGCC-3'	

Rn18s: 18S RNA ribosomal RNA; ***Clec7a***: receptor dectin-1; ***Tlr2***: toll-like receptor 2; ***Tlr4***: toll-like receptor 4.
