# Supplementary material for: Exposition to Biological Control Agent Trichoderma stromaticum Increases the Development of Cancer in Mice Injected With Murine Melanoma
Source: Front Cell Infect Microbiol. 2020 May 29;10:252. doi: 10.3389/fcimb.2020.00252 (PMC7272596; doi:10.3389/fcimb.2020.00252)
Supplement: Supplementary file 2 [file Table_2.pdf]

Supplementary Table 2. Summary of tumor information in experimental mouse model

Group	n	Lung-to-body weight ratio (%)	Tumor uptake n (%)	Visible nodules /mice
Control	6	1.51 ± 0.06	-	-
Conidia	6	1.47 ± 0.06	-	-
B16-F10	6	1.56 ± 0.05	1/6 (17%)	2.66 ± 2.66
Protocol				
PBS + B16-F10	5	1.60 ± 0.06	1/5 (20%)	2 ± 2
Conidia + B16-F10	6	1.60 ± 0.09	6/6** (100%)	13.67 ± 2.56£
Protocol 2				
B16-F10 + PBS	5	1.44 ± 0.05	-/5 (0%)	-
B16-F10 + Conidia	6	1.52 ± 0.04	5/6** (83%)	16.5 ± 5.95£

Data are presented as means ± SEM. Value of $p < 0.05$ were considered for statistical significance. **£:** $p < 0.05$ compared to PBS control of respective protocol by Mann-Whitney test. ****:** $p < 0.01$ compared to PBS control of respective protocol by Chi-square test.
