# Supplementary material for: Exposition to Biological Control Agent Trichoderma stromaticum Increases the Development of Cancer in Mice Injected With Murine Melanoma
Source: Front Cell Infect Microbiol. 2020 May 29;10:252. doi: 10.3389/fcimb.2020.00252 (PMC7272596; doi:10.3389/fcimb.2020.00252)
Supplement: Supplementary file 3 [file Image_1.pdf]

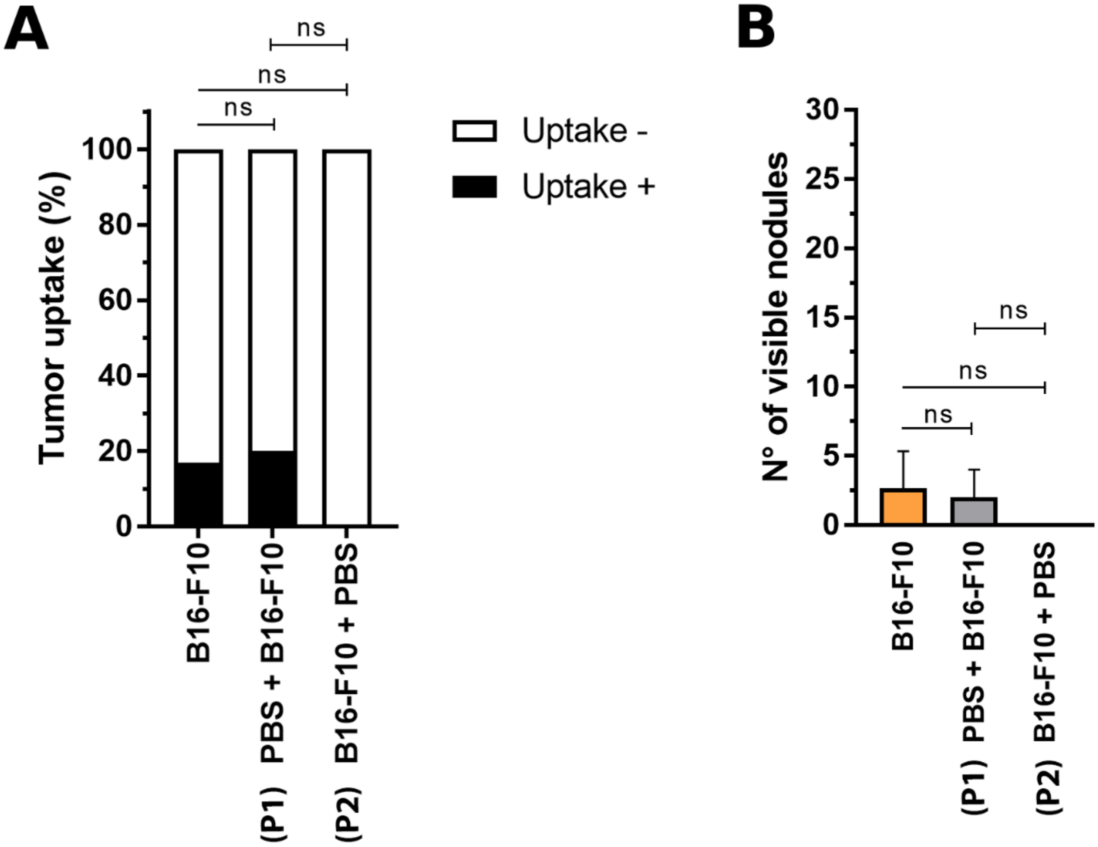

Supplementary Figure S1. Evaluation of tumor development in control groups. The groups were compared for (**A**) tumor uptake using Chi-square test. (**B**) The number of visible nodules in the lung using Kruskal-Wallis test, followed by Dunn's post test. Data are presented as mean ± SEM (n = 5-6 mice per group). Value of $p < 0.05$ was considered for statistical significance.
