# Supplementary material for: Exposition to Biological Control Agent Trichoderma stromaticum Increases the Development of Cancer in Mice Injected With Murine Melanoma
Source: Front Cell Infect Microbiol. 2020 May 29;10:252. doi: 10.3389/fcimb.2020.00252 (PMC7272596; doi:10.3389/fcimb.2020.00252)
Supplement: Supplementary file 4 [file Image_2.pdf]

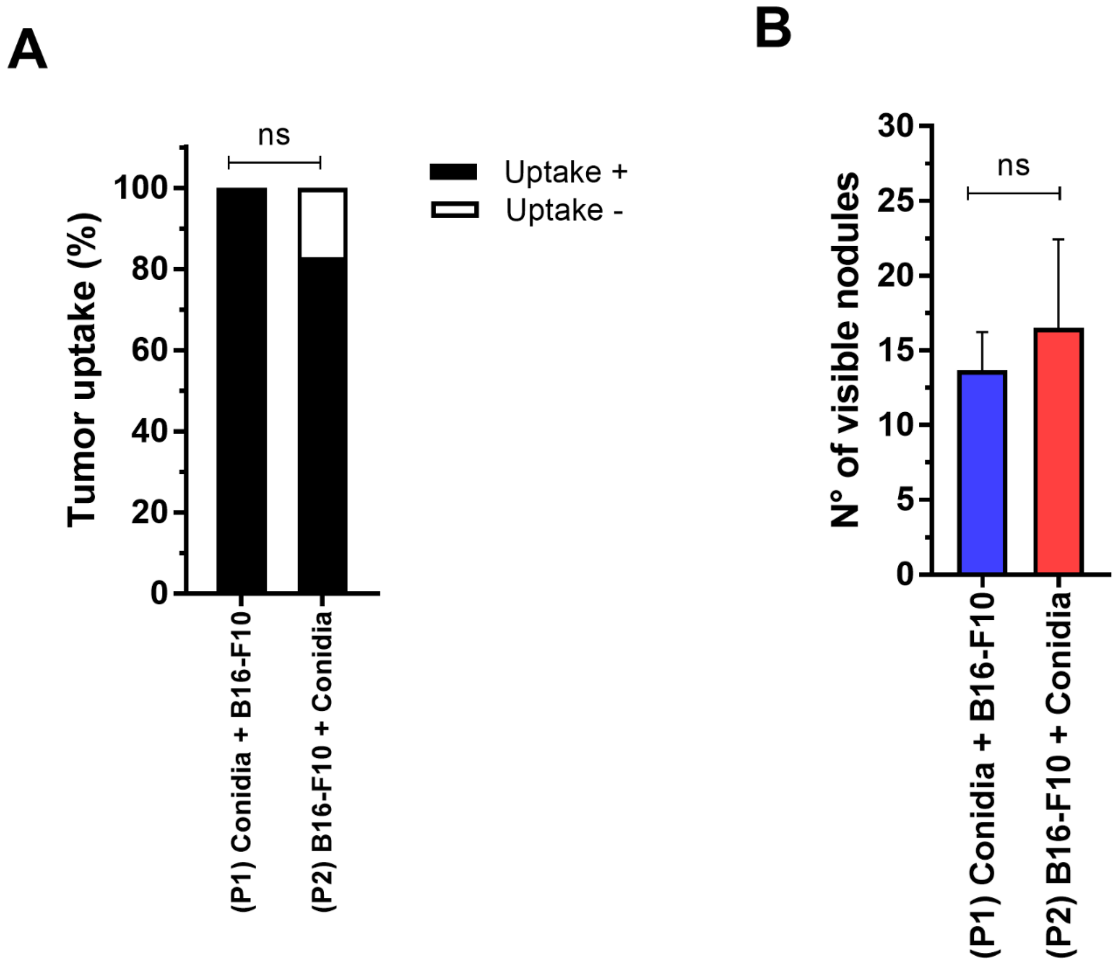

Supplementary Figure S2. Evaluation of tumor development in mice treated with *T. stromaticum* spores from protocol 1 and 2. The groups treated with *T. stromaticum* spores in protocol 1 and 2 were compared to evaluated possible differences between the protocols. (**A**) Tumor uptake was analyzed using Chi-square test. (**B**) Number of visible nodules were analyzed using Mann-Whitney test. Data are presented as mean ± SEM (n = 6 mice per group). Value of $p < 0.05$ was considered for statistical significance.
