# Supplementary material for: Exposition to Biological Control Agent Trichoderma stromaticum Increases the Development of Cancer in Mice Injected With Murine Melanoma
Source: Front Cell Infect Microbiol. 2020 May 29;10:252. doi: 10.3389/fcimb.2020.00252 (PMC7272596; doi:10.3389/fcimb.2020.00252)
Supplement: Supplementary file 5 [file Image_3.pdf]

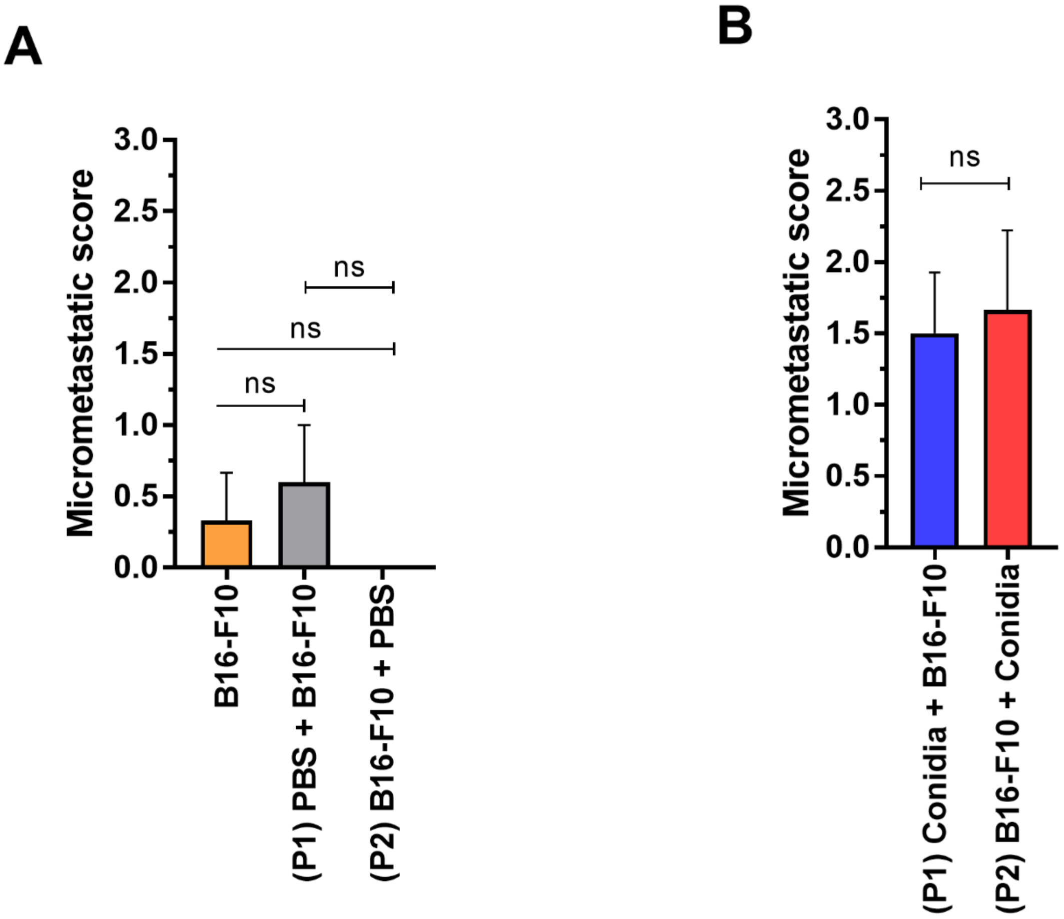

Supplementary Figure S3. Evaluation of micrometastatic score in murine model. (**A**) The control groups B16-F10, PBS+B16-F10 and B16-F10+PBS were compared each other to analyses the micrometastatic score using Kruskal-Wallis test followed by Dunn's post test. (**B**) The groups treated with *T. stromaticum* spores in protocol 1 and 2 were compared to evaluated possible differences between the protocols using Mann-Whitney test. Data are presented as mean ± SEM (n = 5-6 mice per group). Value of $p < 0.05$ was considered for statistical significance.
